# Diffusion weighted hyperpolarized ^129^Xe MRI of the lung with 2D and 3D (FLORET) spiral

**DOI:** 10.1002/mrm.29518

**Published:** 2022-11-09

**Authors:** Abdullah S. Bdaiwi, Matthew M. Willmering, Hui Wang, Zackary I. Cleveland

**Affiliations:** ^1^ Center for Pulmonary Imaging Research, Division of Pulmonary Medicine Cincinnati Children's Hospital Medical Center Cincinnati Ohio USA; ^2^ Department of Biomedical Engineering University of Cincinnati Cincinnati Ohio USA; ^3^ Philips Healthcare Cincinnati Ohio USA; ^4^ Department of Pediatrics University of Cincinnati Cincinnati Ohio USA; ^5^ Imaging Research Center, Department of Radiology Cincinnati Children's Hospital Medical Center Cincinnati Ohio USA

**Keywords:** ADC, diffusion, FLORET, GRE, hyperpolarized ^129^Xe, spiral

## Abstract

**Purpose:**

To enable efficient hyperpolarized ^129^Xe diffusion imaging using 2D and 3D (Fermat Looped, ORthogonally Encoded Trajectories, FLORET) spiral sequences and demonstrate that ^129^Xe ADCs obtained using these sequences are comparable to those obtained using a conventional, 2D gradient‐recalled echo (GRE) sequence.

**Theory and Methods:**

Diffusion‐weighted ^129^Xe MRI (*b*‐values = 0, 7.5, 15 s/cm^2^) was performed in four healthy volunteers and one subject with lymphangioleiomyomatosis using slice‐selective 2D‐GRE (scan time = 15 s), slice‐selective 2D‐Spiral (4 s), and 3D‐FLORET (16 s) sequences. Experimental SNRs from *b*‐value = 0 images ($\mathrm{SNR}0_{\mathrm{EX}}$) and mean ADC values were compared across sequences. In two healthy subjects, a second b = 0 image was acquired using the 2D‐Spiral sequence to map flip angle and correct RF‐induced, hyperpolarized signal decay at the voxel level, thus improving regional ADC estimates.

**Results:**

Diffusion‐weighted images from spiral sequences displayed image quality comparable to 2D‐GRE and produced sufficient $\mathrm{SNR}0_{\mathrm{EX}}$ (16.8 ± 3.8 for 2D‐GRE, 21.2 ± 3.5 for 2D‐Spiral, 20.4 ± 3.5 for FLORET) to accurately calculate ADC. Whole‐lung means and SDs of ADC obtained via spiral were not significantly different (*P* > 0.54) from those obtained via 2D‐GRE. Finally, 2D‐Spiral images were corrected for signal decay, which resulted in a whole‐lung mean ADC decrease of ˜15%, relative to uncorrected images.

**Conclusions:**

Relative to GRE, efficient spiral sequences allow ^129^Xe diffusion images to be acquired with isotropic lung coverage (3D), higher SNR(2D and 3D), and three‐fold faster (2D) within a single breath‐hold. In turn, shortened breath‐holds enable flip‐angle mapping, and thus, allow RF‐induced signal decay to be corrected, increasing ADC accuracy.

## 1 INTRODUCTION

Hyperpolarized (HP) gas MRI using ^3^He and ^129^Xe has emerged as a powerful, noninvasive modality to quantify regional lung function and structure,^1^, ^2^, ^3^, ^4^, ^5^, ^6^ with ^129^Xe increasingly used due to greater abundance and improved hyperpolarization technology.^7^, ^8^ Furthermore, diffusion‐weighted HP gas MRI—specifically ADC mapping—has become a validated tool to assess the dimensions of pulmonary microstructure.^9^, ^10^, ^11^, ^12^, ^13^, ^14^, ^15^ Once inhaled, hyperpolarized gas atoms diffuse a smaller distance, relative to free diffusion, because of collisions with alveolar walls. However, diffusion restriction decreases as airspace‐size increases, leading to larger ADCs. As such, hyperpolarized gas ADC can probe alveolar size during normal development^11^, ^14^, ^16^, ^17^ and lung‐disease induced remodeling, as occurs in idiopathic pulmonary fibrosis (IPF),^15^, ^18^, ^19^ lymphangioleiomyomatosis (LAM),^20^ and chronic obstructive pulmonary disease (COPD).^11^, ^15^, ^21^ Thus, ^129^Xe diffusion MRI may become a useful clinical tool to evaluate and monitor lung disease.

The ^129^Xe ADC is commonly measured using slice‐selective, 2D gradient recalled echo (GRE) sequences with breath‐hold period of up to 16 s.^11^, ^14^, ^15^ These relatively long breath‐holds are difficult for poorly compliant subjects (e.g., children) and patients with compromised respiratory function. Moreover, they can be insufficient to enable full lung coverage at high resolution, particularly when acquiring multiple *b*‐values to quantify airspace morphometry.^12^, ^22^ Therefore, diffusion‐weighted ^129^Xe MR images are often obtained with modest slice numbers,^5^, ^6^, ^7^, ^8^, ^9^, ^10^ thick slices (15–30 mm), or gaps (5 mm or more) between slices.^11^, ^12^, ^14^ Because alveolar remodeling is spatially heterogeneous and often focal in early lung disease,^20^, ^23^ incomplete lung coverage may bias results and miss early signs of disease.

Compared to ^3^He, diffusion‐weighted MRI with HP ^129^Xe has been more challenging due to its three‐fold lower gyromagnetic ratio,^1^ historically lower polarizations (˜10%),^3^, ^11^ and six‐fold lower diffusivity.^1^ These complications necessitated longer diffusion gradients, echo times, and repetition times that degrade SNR and reduce the accuracy of ADC measurements.^14^, ^24^ SNR is further reduced in diffusion‐weighted HP ^129^Xe imaging with 2D‐GRE sequences due to T_1_ relaxation during the breath‐hold and depolarization caused by the relatively large number of RF pulses (40–120 per slice) needed for Cartesian k‐space sampling.

Spiral sequences sample k‐space with high efficiently, making them well‐suited for HP gases imaging, because they reduce T_1_ losses and require only ˜10 polarization‐destroying RF pulses for 2D acquisitions^25^, ^26^, ^27^, ^28^ and ˜280 for isotropic, 3D acquisitions.^25^ High sampling efficiency allows the use of larger flip angles (α) to increase HP image SNR without consuming all of the available non‐equilibrium magnetization.^1^ However, if sufficiently large flip angles are used, RF‐induced signal decay will become comparable to the signal attenuation caused by diffusion weighting, leading to systematically and spuriously increased ADC estimates unless images are corrected for regional signal decay.

Here we demonstrate ^129^Xe diffusion imaging using 2D and 3D (**F**ermat **L**ooped, **OR**thogonally **E**ncoded **T**rajectories, FLORET^29^, ^30^) spiral sequences. The 3D implementation provides isotropic (5 mm^3^ × 5 mm^3^ × 5 mm^3^), whole‐lung coverage within a conventional breath‐hold (≤16 s), whereas the 2D implementation enables rapid acquisitions (3 *b*‐values in <5 s). Finally, we demonstrate a method to acquire diffusion images and flip angle maps during the same breath‐hold using 2D‐Spiral. These maps allow B_1_ inhomogeneity and RF induced signal decay, thus improve the accuracy of HP ^129^Xe ADC measurements.

## 2 THEORY

### 2.1 SNR

The HP ^129^Xe magnetization, $M_{\mathrm{hp}}$, available at clinical field strengths, exceeds that of thermally polarized ^129^Xe by approximately five orders of magnitude.^1^, ^3^, ^6^ Thus, $M_{\mathrm{hp}}$ can be assumed to decay monotonically to its thermal equilibrium value, M(0), due solely to T_1_ relaxation and RF depletion.^1^, ^6^ When using a constant α the signal arising from the *nth* RF pulse $\left(n=1,2\ldots N_{\mathrm{RF}}\right)$ can therefore be written as:

$$\begin{array}{ll} S(n) & =f\;M_{\mathrm{hp}}(0)\;\sin(\alpha ){\left(\cos(\alpha )\;\exp\left(-\frac{\mathrm{TR}}{T_{1}}\right)\right)}^{n-1}\\ & \;\times \exp\left(-\frac{\mathrm{TE}}{T_{2}^{*}}\right), \end{array}$$

(1) $$=f\;M_{\mathrm{hp}}(0)\;\sin(\alpha )C_{1}^{n-1}\;\exp\left(-\frac{\mathrm{TE}}{T_{2}^{*}}\right),\;$$

where f is a system and volume specific sensitivity factor, TR is the repetition time, TE is the echo time, $T_{1}$ is the longitudinal relaxation time (˜30 s in the lung at 3 T^31^, ^32^), $T_{2}^{*}$ is the apparent transverse relaxation time (18 ms in human lung at 3T^33^), and $C_{1}=\cos\left(\alpha \right)\exp\left(-\frac{\mathrm{TR}}{T_{1}}\right)$.

For ^129^Xe diffusion imaging using GRE and linear encoding, diffusive signal attenuation resulting from the *wth* diffusion weighting (w=1,2…W) can be modeled using Eq. 1 and the Stejskal–Tanner model^34^as:

(2) $$\begin{array}{ll} S\left(n_{\mathrm{ph}},w\right) & =fM_{\mathrm{hp}}(0)\;\sin(\alpha )\;e^{-b_{w}\mathrm{ADC}}C_{1}^{W\left(n_{\mathrm{ph}}-1\right)}C_{1}^{\left(w-1\right)}\\ & \;\times \exp\left(-\frac{\mathrm{TE}}{T_{2}^{*}}\right), \end{array}$$

where $n_{\mathrm{ph}}$ is the index of the phase encoding ($n_{\mathrm{ph}}=1,2\ldots N_{\mathrm{ph}}$), $N_{\mathrm{ph}}$ is the total number of phase encoding steps for a given slice/volume, and $b_{w}$ is the gradient time and amplitude‐dependent parameter (i.e., *b*‐value) that describes magnitude of the diffusion weighting.

Because the signal magnitude for a Cartesian acquisition is dominated by the central line in k‐space, k_0_, the theoretical SNR of the $b_{w}$ = 0 image ($\mathrm{SNR}0_{\mathrm{TH}}$) can be defined for fixed, 2D acquisition matrix with linear phase encoding as

(3) $$\begin{array}{ll} \mathrm{SNR}0_{\mathrm{TH}}(\mathrm{GRE}) & =\frac{S\left(n_{\mathrm{ph}(0)}\right)}{\text{noise}}\\ & =f\frac{M_{\mathrm{hp}}(0)\;\sin(\alpha )C_{1}^{n_{\mathrm{ph}(0)}-1}\;\exp\left(-\frac{\mathrm{TE}}{T_{2}^{*}}\right)}{\sigma_{S}/\sqrt{N_{\mathrm{ph}}T_{s}}}, \end{array}$$

where $\sigma_{S}$ is the SD of the noise, $T_{s}$ is the sampling time per line of k‐space, $\sigma_{S}/\sqrt{N_{\mathrm{ph}}T_{s}}$ is the total noise contribution to Eq. 3. Furthermore, $n_{\mathrm{ph}\left(0\right)}=\frac{N_{\mathrm{RF}}}{2}+1$, where $N_{\mathrm{RF}}=W\times N_{\mathrm{ph}}$ is the total number of excitations, and W is the total number of diffusion weightings.

For center‐out trajectories such as spiral, k_0_ is sampled as the first data point in each k‐space view, and image signal can be approximated as being proportional to the average magnitude of all contributing k_0_ points. If data are encoded such that all *b*‐values are acquired in ascending order before progressing to the next k‐space trajectory, the signal intensity for a given spiral view ($n_{s}=1,2\ldots N_{s}$) is given by^35^

$$\begin{array}{ll} S\left(n_{s},w\right) & =f\frac{M_{\mathrm{hp}}(0)\sin(\alpha )}{N_{s}}e^{-b_{w}\mathrm{ADC}}\sum_{n_{s}=1}^{N_{s}}C_{1}^{W\left(n_{s}-1\right)}C_{1}^{\left(w-1\right)}\;\\ & \;\times \exp\left(-\frac{\mathrm{TE}}{T_{2}^{*}}\right) \end{array}$$

(4) $$\begin{array}{ll} & \;=f\frac{M_{\mathrm{hp}}(0)\sin(\alpha )}{N_{s}}e^{-b_{w}\mathrm{ADC}}C_{1}^{\left(w-1\right)}\left[\frac{1-C_{1}^{W\cdot N_{s}}}{1-C_{1}^{W}}\right]\\ & \;\times \exp\left(-\frac{\mathrm{TE}}{T_{2}^{*}}\right). \end{array}$$

$\mathrm{SNR}0_{\mathrm{TH}}$ ($b_{w}=0,w=1$) can then be defined as

(5) $$\begin{array}{cc} & \mathrm{SNR}0_{\mathrm{TH}}(\text{Spiral})=\frac{S\left(N_{s}\right)}{\text{noise}}\\ & \;=\frac{f}{N_{s}}\frac{M_{\mathrm{hp}}(0)\;\sin(\alpha )\left[\frac{1-C_{1}^{W\cdot N_{s}}}{1-C_{1}^{W}}\right]\exp\left(-\frac{\mathrm{TE}}{T_{2}^{*}}\right)}{\sigma_{S}/\sqrt{N_{s}T_{s}}}, \end{array}$$

where $N_{s}$ is the number of spirals needed to encode a given 2D slice or 3D volume. Note, the contribution of the sampling efficiency (i.e., non‐uniform sampling) to SNR for spiral is expected to be small^30^, ^36^ and is neglected in Eqs. 4 and 5. To allow SNR comparisons across acquisition types, voxel volume can be incorporated into Eqs. 3 and 5 according to^37^, ^38^:

(6) $$fM_{\mathrm{hp}}\propto \text{ΔxΔyΔz}$$

where ΔxΔy, and Δz are the voxel dimensions.

### 2.2 Regional magnetization decay correction

For center‐out sampling methods (i.e., spiral), if flip angles are sufficiently large (often used in 2D‐Spiral), RF‐induced signal decay across *b*‐values will become comparable to the signal attenuation caused by diffusion weighting. This is expected to systematically and spuriously increase the calculated ADC. Thus, mapping flip angle and correcting for RF‐induced signal decay are required to improve the accuracy of the ADC estimate. Flip angle maps can be generated if the first and last diffusion weightings (i.e., w = 1 and w = W) are acquired with $b_{w}$ = 0 s/cm^2^. If the acquisition time is ≪T_1_, which is satisfied for 2D spiral acquisitions, a flip angle map can be obtained from Eq. 4 by calculating the voxel‐by‐voxel ratio of the first *b*‐value = 0 image, $S_{1}$, and the second *b*‐value = 0 image, $S_{W}$, according to

(7) $$\frac{S_{W}}{S_{1}}={\left[\cos\left(\alpha_{\text{local}}\right)\right]}^{\left(W-1\right)}.$$

Solving for the $\alpha_{\text{local}}$ yields

(8) $$\alpha_{\text{local}}=\arccos\left(\sqrt[{W-1}]{\frac{S_{W}}{S_{1}}}\right).$$

To correct for RF induced decay and B_1_ inhomogeneity, magnitude images can then be multiplied by the factor

(9) $$N_{s}\frac{1}{\alpha_{\text{local}}}\frac{1}{\sin\left(\alpha_{\text{local}}\right)}\frac{1}{\cos{\left(\alpha_{\text{local}}\right)}^{w-1}}\left[\frac{1-\cos{\left(\alpha_{\text{local}}\right)}^{W}}{1-\cos{\left(\alpha_{\text{local}}\right)}^{N_{s}W}}\right],$$

where the factor of $1/\alpha_{\text{local}}$ has been included to account for the receive sensitivity of the RF coil.^39^, ^40^

## 3 METHODS

### 3.1 $\mathrm{SNR}0_{\mathrm{TH}}$ Evaluation

Using Eqs. 3, 5, and 6, the $\mathrm{SNR}0_{\mathrm{TH}}$ were evaluated using MATLAB‐2020b (The MathWorks, Inc., Natick, MA) across a range of parameters given in Table 1 for Cartesian GRE and spiral sampling methods. For spiral sequences, Archimedean spiral with uniform density and linear ordering was assumed. For simplicity, $\sigma_{S}$ was held constant across sequences.

**TABLE 1:** Acquisition parameters used for calculating $\mathrm{SNR}0_{\mathrm{TH}}$ for the three sequences

Parameters	2D‐GRE	2D‐Spiral	3D‐FLORET
$f\cdot M_{\mathrm{hp}}\left(0\right)$	375	375	125
TE (ms)	10	8	7
T_s_ (ms)	3.7	10	10
TR (ms)	14	19	18
Excitations	N_ph_ = 56	N_s_ = 10	N_s_ = 280
$V_{\mathrm{vox}}$ (mm^3^)	5 × 5 × 15	5 × 5 × 15	5 × 5 × 5
W	3	3	3
Flip Angle (^o^)	0.5–30	0.5–30	0.5–30
$T_{2}^{*}$ (ms) at 3 T	18	18	18
T_1_ (s) at 3 T	30	30	30
$\sigma_{S}$	50	50	50

### 3.2 In vivo Image Acquisition

Human studies were approved by the Cincinnati Children's Research Foundation, Institutional Review Board (IRB) and the U.S. Food and Drug Administration via an investigational new drug application (IND 123577). HP ^129^Xe diffusion MRI was performed in 4 healthy volunteers (5 y, male; 12, male; 25, male; and 47, female) and 1 patient with LAM (51 y, female). Informed consent was obtained from adult participants. For pediatric participants, parental consent and age‐appropriate assent was obtained. Isotopically enriched ^129^Xe (85% ^129^Xe, Linde Elec. & Specialty Gasses Inc., Alpha, NJ) was polarized to 20%–40% using a commercial polarizer (Model 9820, Polarean Imaging, plc, Durham, NC). Polarized ^129^Xe was cryogenically collected under liquid nitrogen and thawed into Tedlar bags (Jensen Inert Products, Coral Springs FL). The xenon dose per image was 1 L for adults and 1/6th of pediatric subjects' total lung capacity (maximum of 1 L), estimated according to pediatric ATS plethysmography‐based guidelines.^41^

Images were acquired with a 3T Philips Ingenia MRI scanner (Philips Healthcare, Best, Netherlands), (maximum gradient amplitude = 21 mT/m; maximum gradient slew rate = 200 mT/m/ms) and a flexible transmit/receive ^129^Xe chest coil (Clinical MR Solutions, Brookfield, WI, USA). Subjects were imaged in the supine position, and conventional breath‐hold proton images were acquired to localize subjects for ^129^Xe acquisitions. Subjects exhaled to functional residual capacity (FRC) and inhaled HP ^129^Xe prior to breath‐holds of ≤16 s. The first dose (˜25% xenon diluted 75% with ultrapure N_2_) was used to calibrate the global flip angle and determine ^129^Xe resonance frequency. Subsequent doses, containing pure xenon, were used for diffusion imaging (2D‐GRE, 2D‐Spiral, then FLORET).

**FIGURE 1:**
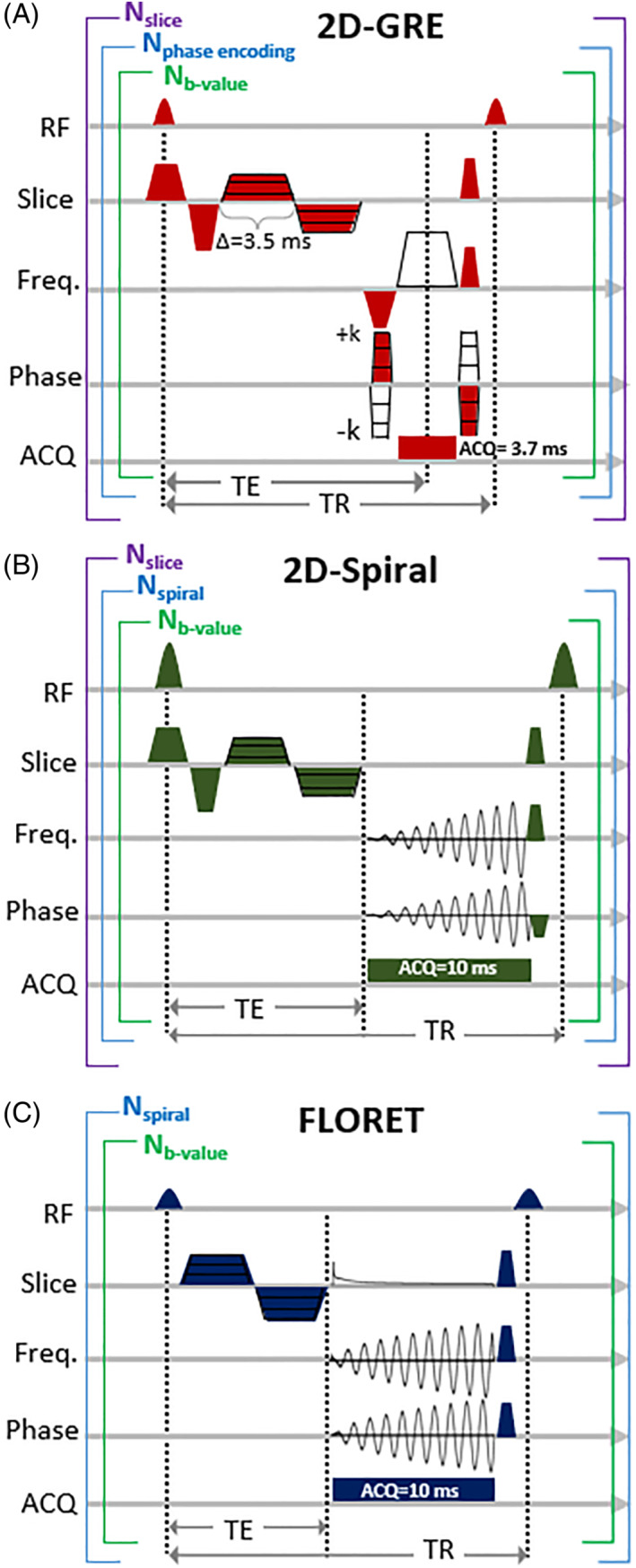
^129^Xe diffusion sequences with bipolar diffusion encoding gradients (Δ = δ = 3.5 ms) placed between excitation and acquisition. *b*‐values were acquired sequentially (*b* = 0 to *b*_max_, inner green loop) before progressing to the next line of k‐space (middle blue loop). A, 2D‐GRE pulse sequence. All lines of k‐space for all *b*‐values were acquired before progressing to the next slice (outer purple loop). B, 2D‐Spiral sequence. Archimedean spiral (uniform density) readout was implemented with the same diffusion encoding and acquisition loop order as (A). C, FLORET using one‐hub (linear ordering). *b*‐values were acquired first (green inner loop) before moving to the next projection (blue outer loop).

Diffusion‐weighing used bipolar diffusion encoding gradients (diffusion time, Δ = 3.5 ms) placed between excitation and data acquisition (Figure 1). For spiral sequences, Archimedean spiral (uniform density) was used. For FLORET, a single hub was used where each spiral is projected onto a unique cone (between +90° and − 90°) to fully acquire k‐space. MRI parameters included: 3 *b*‐values = 0–15 s/cm^2^; field‐of‐view = 320 × 280 mm^2^, 320 × 320 mm^2^, and 320 × 320 × 320 mm^3^ for 2D‐GRE, 2D‐Spiral, and FLORET, respectively; voxel size = 5 mm × 5 mm × 15 mm (2D‐GRE and 2D‐Spiral), 5 mm × 5 mm × 5 mm (FLORET); coronal slices = 6 (2D‐GRE and 2D‐Spiral) and = 64 (FLORET); and scan time = 15 s (2D‐GRE), = 4 s (2D‐Spiral), and 16 s (FLORET). A full list of acquisition parameters is given in Table 2. Linear phase encoding in the apical‐to‐caudal direction was used for 2D‐GRE sequence. For a given set of k‐space trajectories, all *b*‐values were acquired sequentially (ascending order) before progressing to the next Cartesian phase‐encoding line or spiral trajectory. Slices were acquired in the anterior‐to‐posterior direction, and all k‐space lines (phase encodes or spirals) and all *b*‐values were acquired before progressing to the next slice (Figure 1).

**TABLE 2:** In vivo acquisition parameters for 2D‐GRE, 2D‐Spiral and FLORET sequences

Acquisition parameters	2D‐GRE	2D‐Spiral	3D‐FLORET
*b*‐values (s/cm^2^)	0, 7.5, 15	0, 7.5, 15	0, 7.5, 15
		0, 7.5, 15, 0*	
TE (ms)	10.2	8.0	7.15
TR (ms)	13.3	19.3	19.2
Readout (ms)	3.7	10	10
Excitations	50–75/slice/*b*‐value	5–10/slice/*b*‐value	270–290/*b*‐value
Encoding method	Linear: +ky to ‐ky	Archimedean spiral: uniform density	One‐hub: linear ordering
Voxel size (mm)	5 × 5 × 15 (gap = 15)	5 × 5 × 15 (gap = 15)	5 × 5 × 5 (no gap)
Matrix size	64 × 56	64 × 64	64 × 64 × 64
Number of slices	5–6	6	64
Flip angle	5.6–9°	15–25°	3–4.5°
Scan time (s)	15	4	16
Scan time per voxel volume (ms/mm^3^)	0.69	0.16	0.06

* Second b = 0 s/cm^2^ images was used for RF decay and B_1_ inhomogeneity correction.

The global flip angle was prescribed depending on $N_{\mathrm{RF}}$ to ensure the last *b*‐value image has sufficient SNR for accurate ADC measurements, according to.^14^

(10) $$\alpha_{\text{global}}=\cos^{-1}\left[{\left(0.2\right)}^{\frac{1}{N_{\mathrm{RF}}}}\right],$$

Eq. 10 (neglecting T_1_) ensures 20% of the magnetization remains after acquisition. $N_{\mathrm{RF}}=N_{\mathrm{ph}}\times W$ for Cartesian and $N_{S}\times W$ for spiral acquisition.

### 3.3 Image reconstruction

All Images were reconstructed offline using a Graphical Programming Interface (GPI).^42^ 2D‐GRE images were reconstructed by applying 2D Fourier transformation (FT). For 2D‐Spiral and FLORET, all points along a projection were scaled to the mean k_0_ across spiral interleaves prior to reconstruction to reduce image artifacts from global signal decay.^25^, ^43^ Scaled data were re‐grided into a Cartesian matrix using the default GPI iterative density compensation and gridding settings followed by 2D FT for 2D‐Spiral or 3D FT for FLORET.^44^ Further image and data analysis was performed in MATLAB‐2020b.

### 3.4 Image Analysis

Binary lung masks were created by manually segmenting ventilated lung volume (excluding airways) in the *b*‐value = 0 (b_0_) images. A region of interest consisting of all unventilated background within the image (i.e., excluding lungs, large airways, and visually obvious artifacts) was used to calculate noise. The experimental SNR of the $b_{w}$ = 0 image ($\mathrm{SNR}0_{\mathrm{EX}}$) was calculated according to $\left(S_{\text{lung}}-S_{\mathrm{BG}}\right)/\sigma_{\mathrm{BG}}$, where $S_{\text{lung}}$ is mean signal amplitude within the mask; $S_{\mathrm{BG}}$ is the mean amplitude of the background noise; and $\sigma_{\mathrm{BG}}$ is the SD of the noise. $\sigma_{\mathrm{BG}}$ was calculated from the noise measured in the background, $\sigma_{M}$, according to ${\sigma_{\mathrm{BG}}^{2}=\sigma }_{M}^{2}/\left(2-\pi /2\right).$ ^14^, ^24^, ^45^ To allow SNR comparisons across sequences and HP ^129^Xe doses, voxel volume, $V_{\mathrm{vox}}$; ^129^Xe polarization, $P_{\mathrm{Xe}}$; and ^129^Xe dose volume, $V_{\mathrm{Xe}}$ were considered by normalizing $\mathrm{SNR}0_{\mathrm{EX}}$ according to

(11) $$\mathrm{NSNR}0_{\mathrm{EX}}=\frac{\bar{\mathrm{SNR}0_{\mathrm{EX}}}}{V_{\mathrm{vox}}xP_{\mathrm{Xe}}xV_{\mathrm{Xe}}},$$

where $\bar{\mathrm{SNR}0_{\mathrm{EX}}}$ is the average$\mathrm{SNR}0_{\mathrm{EX}}$ across ventilated voxels.

To mitigate RF decay globally and allow signal comparisons across sequences, diffusion‐weighted images from all sequences were scaled by $S_{w}/S_{w=1}\cos{\left(\alpha_{\text{global}}\right)}^{w-1}$, where $S_{w}$ is the diffusion‐weighted signal for a given *b*‐value $\left(b_{w}\right)$, and w is the index of *b*‐values (w=1,2,3 for $b_{w}=0,7.5,15$ s/cm^2^, respectively), prior to reconstruction.^17^ However, this global correction was omitted when voxel‐level flip angle correction was used (section 3.5). ADC was calculated voxel‐by‐voxel using log‐linear fitting according to^14^

(12) $$\mathrm{ADC}=-\frac{W\sum_{w=1}^{W}b_{w}\ln\left(S_{w}\right)-\sum_{w=1}^{W}b_{w}\sum_{w=1}^{W}\ln\left(S_{w}\right)}{W\sum_{w=1}^{W}b_{w}^{2}-{\left(\sum_{w=1}^{W}b_{w}\right)}^{2}}.$$

### 3.5 Voxel‐Level RF Induced Decay Correction (2D‐Spiral)

To obtain the flip angle maps, 2D‐Spiral scanning was performed with *b*‐value = 0, 7.5, 15, and 0 s/cm^2^ in 2 of healthy subjects (ages 5 and 12 y). Using the second b = 0 s/cm^2^ image, flip angle maps were calculated directly from voxel‐level intensities according to Eq. 8. Maps were then smoothed using a 2D Gaussian smoothing kernel with SD of 1. Magnitude images were multiplied by the correction factor given by Eq. 9.

### 3.6 Statistical Analysis

$\mathrm{SNR}0_{\mathrm{EX}}$, $\mathrm{NSNR}0_{\mathrm{EX}}$, whole‐lung ADC means, SDs and coefficients of variation (CVs) were calculated for each subject and pulse sequence. $\mathrm{SNR}0_{\mathrm{EX}}$, $N{\mathrm{SNR}}_{\mathrm{EX}}$ and ADC means and SDs were compared across all sequences using a Wilcoxon rank sum test with adjusting the significant level to account for type 1 error (significant level = 0.05/3 = 0.0166). Additionally, mean ADC was correlated with healthy subject age excluding the LAM subject for each sequence via linear regression.

## 4 RESULTS

### 4.1 Theoretical SNR $\left(\mathrm{SNR}0_{\mathrm{TH}}\right)$

**FIGURE 2:**
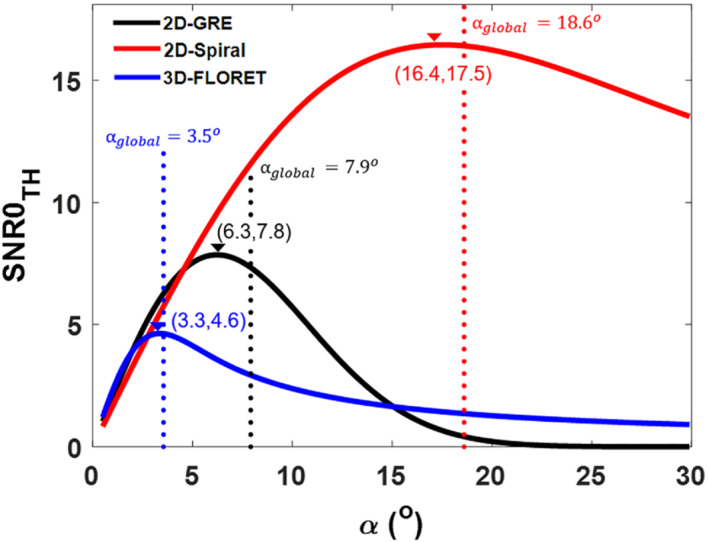
Theoretical signal‐to‐noise ratio, $\mathrm{SNR}0_{\mathrm{TH}}$ as a function of the flip angle, α, using parameters in Table 1 and Eq. 3 (2D‐GRE) and Eq. 5 (2D‐Spiral and FLORET). For each sequence, $\mathrm{SNR}0_{\mathrm{TH}}$ is expected to be maximized at specific flip angle (color‐matched triangles above curves), with FLORET displaying decreased $\mathrm{SNR}0_{\mathrm{TH}}$, relative to the 2D‐GRE. 2D‐Spiral is expected to produce substantially higher $\mathrm{SNR}0_{\mathrm{TH}}$ than either 2D‐GRE or FLORET. For all three sequences, the maximum achievable $\mathrm{SNR}0_{\mathrm{TH}}$ only slightly exceeded (i.e., <4%) the signal expected using $\alpha_{\text{global}}$ as defined in Eq. 10 (dashed vertical lines).

The dependence of $\mathrm{SNR}0_{\mathrm{TH}}$ on flip angle is shown in Figure 2. For a given number of RF pulses (see Table 1), a flip angle exists at which $\mathrm{SNR}0_{\mathrm{TH}}$ is maximized (marked peaks, triangles over curve) for each sequence. Depending on matrix size, 2D‐GRE requires 40–120 RF pulses per slice to fully sample *k*‐space. Therefore, modest flip angles must be applied (5–10°) to preserve HP magnetization for all k‐space lines. However, 2D‐Spiral requires only 5–20 RF pulses to yield comparable resolution. Reduced RF pulse number allows larger flip angle to be used without excessively depleting HP magnetization, resulting in higher$\mathrm{SNR}0_{\mathrm{TH}}$ relative to 2D‐GRE and FLORET. In contrast, FLORET requires the use of relatively small flip angles (3–5°) to preserve magnetization over 270–290 RF excitations. FLORET is also an isotropic, 3D sequence, which enables smaller voxel sizes compared to the 2D sequences (see Table 1). A combination of these two factors (small flip angles and reduced voxel size) is expected to decrease $\mathrm{SNR}0_{\mathrm{TH}}$ values from FLORET relative to 2D‐GRE (by nearly one‐fold) and 2D‐Spiral (by greater than three‐fold).

Another factor to consider that effects the $\mathrm{SNR}0_{\mathrm{TH}}$ and SNR in general is the sampling time. For spiral acquisitions, the sampling time was 10 ms, relative to the 3.7 ms for 2D‐GRE, and this increases the SNR by a factor of $\sqrt{T_{s}}$ (see Eq. 5). Therefore, the significant increase in $\mathrm{SNR}0_{\mathrm{TH}}$ in 2D‐Spiral sequence compared to the 2D‐GRE results from higher sampling efficiency allowing larger applied flip angles and longer sampling times.

The flip angles values that maximize $\mathrm{SNR}0_{\mathrm{TH}}$ (marked peaks in Figure 2) were lower (by 22%, 12% and 6% for 2D‐GRE, 2D‐Spiral, and FLORET, respectively) compared to the $\alpha_{\text{global}}$ generated using Eq. 10. However, the expected signal intensity variations are relatively flat for flip angles within ˜15% of optimal, therefore RF‐dependent SNR variations will be minimal for most flip‐angle‐selection schemes.^39^ For 2D‐GRE, the $\alpha_{\text{global}}$ is 22% higher than predicted by Eq. 3. Note, this difference is expected, because Eq. 10 is designed to retain 20% of HP magnetization after data acquisition.

### 4.2 In Vivo Image Quality

**FIGURE 3:**
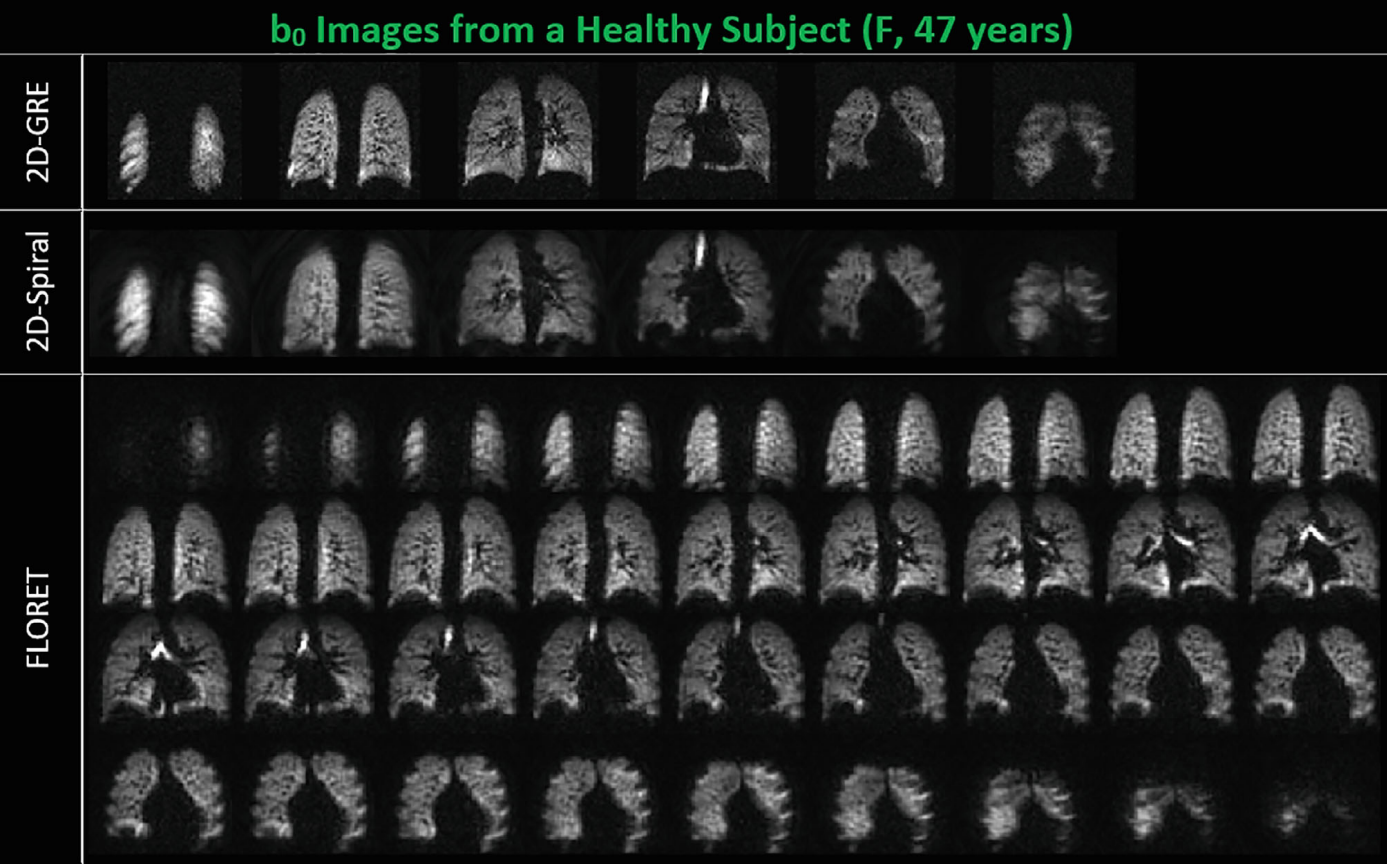
Comparison of b_0_ images from a healthy female volunteer (47 y). Each row corresponds to the sequences used: *top*, 2D‐GRE; *middle*, 2D‐Spiral; and *bottom*, FLORET. Scan time was 15 s (0.69 ms per voxel) for 2D‐GRE, 4 s (0.16 ms per voxel) for 2D‐Spiral and 16 s (0.06 ms per voxel) for FLORET. Images obtained using all sequences showed qualitatively good agreement in depicting structural features.

The b_0_ images from a healthy subject and each sequence are shown in Figure 3. Both 2D and 3D spiral sequences display image quality (e.g., SNR and resolvable features) that are comparable to 2D‐GRE images. In addition, no significant susceptibility‐induced image degradation (i.e., signal loss and geometric distortion) was evident for either type of spiral image. However, there was small degree of blurring in 2D‐Spiral images, which likely resulted from the 2.7 times longer readout, relative to 2D‐GRE.

**FIGURE 4:**
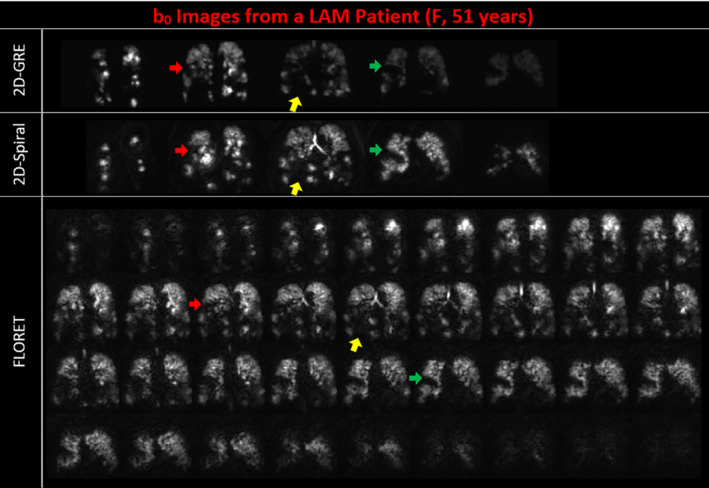
Comparison of b_0_ images from a subject with LAM (female, 51 y). Row corresponds to the sequences used: top, 2D‐GRE; middle, 2D‐Spiral; and bottom, 3D FLORET. Scan time was 15 s (0.69 ms per voxel) for 2D‐GRE, 4 s (0.16 ms per voxel) for 2D‐Spiral and 16 s (0.06 ms per voxel) for FLORET. All sequences showed minimal image artifacts and good correlation of structural features. For example, ventilation defects (red, yellow and green arrows) were consistently observed in the same regions.

Similar overall patterns in image quality are seen in Figure 4, which shows b_0_ images of the LAM patient. Aside from minor blurring in 2D‐Spiral images, spiral sequences again provide comparable images to 2D‐GRE. Ventilation defects (e.g., red, yellow, and green arrows) are visible in all images and are located predominantly in the middle/lower portions of the lungs. This heterogeneity is captured across all sequences, with ventilation defects consistently observed in the same anatomical regions. While subtle differences are present, they can likely be attributed to variations in ventilation distribution between breath‐holds and small differences in subject position.

### 4.3 Experimental SNR $\left(\mathrm{SNR}0_{\mathrm{EX}}\text{and}\mathrm{NSNR}0_{\mathrm{EX}}\right)$

**FIGURE 5:**
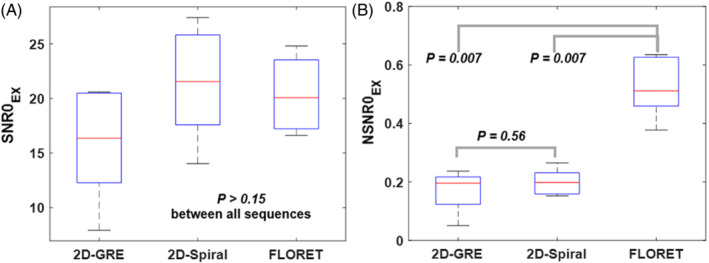
$\mathrm{SNR}0_{\mathrm{EX}}$ and $\mathrm{NSNR}0_{\mathrm{EX}}$ across subjects and sequences. A, $\mathrm{SNR}0_{\mathrm{EX}}$ showed no significant difference between the three sequences (*P > 0.15*). B, No significant difference in $\mathrm{NSNR}0_{\mathrm{EX}}$ was observed between the 2D sequences (*P = 0.56*). However, FLORET displayed a significant increase (˜2.5‐fold) in $\mathrm{NSNR}0_{\mathrm{EX}}$ over both 2D‐GRE and 2D‐Spiral (*P = 0.007*).

While 2D images had acceptable in‐plane resolution (5 × 5 mm^2^), they were obtained with 15 mm gaps between slices and three‐fold thicker slices than the FLORET image (5 mm isotropic). Mean experimental $\mathrm{SNR}0_{\mathrm{EX}}$ was comparable and sufficiently high (16.8 ± 3.8 for 2D‐GRE, 21.2 ± 3.5 for 2D‐Spiral, and 20.4 ± 3.5 for FLORET) to permit accurate ADC estimation across subjects and sequence types (Figure 5A). However, because $\mathrm{SNR}0_{\mathrm{EX}}$ does not account for the difference in ^129^Xe polarization between doses or voxel volume, $\mathrm{NSNR}0_{\mathrm{EX}}$ enables a more informative comparison across sequence types. Mean experimental $\mathrm{NSNR}0_{\mathrm{EX}}$ was 0.16 ± 0.04 for 2D‐GRE, 0.19 ± 0.05 for 2D‐Spiral and 0.54 ± 0.10 for FLORET (Figure 5B). The FLORET sequence provides significantly higher $\mathrm{NSNR}0_{\mathrm{EX}}$ (˜2.5‐fold increase) relative to the 2D‐GRE and 2D‐Spiral (*P* < 0.007).

This trend result agrees with a previous study showing an SNR gain of 1.4‐fold in peripheral lung and 1.7‐fold in airways when using 3D‐GRE, relative to 2D‐GRE in ^129^Xe ventilation imaging.^46^ The SNR improvement was attributed to the lack of diffusive dephasing during slice selection for 3D sequences. However, the experimental $\mathrm{NSNR}0_{\mathrm{EX}}$ for FLORET (see Figure 5) produced significantly higher $\mathrm{NSNR}0_{\mathrm{EX}}$, compared to those predicted by the theoretical model (Eqs. (3), (4), (5), (6), Figure 2). This results primarily from FLORET producing lower image noise levels compared to the 2D sequences, with the SD of the background noise is reduced by 35% average across subjects. Additionally, 2D‐Spiral produced lower $\mathrm{NSNR}0_{\mathrm{EX}}$ values than predicted. These disagreements could result from the simple theoretical model neglecting noise‐like contributions resulting from image acquisition and reconstruction imperfections.

### 4.4 ADC maps

**FIGURE 6:**
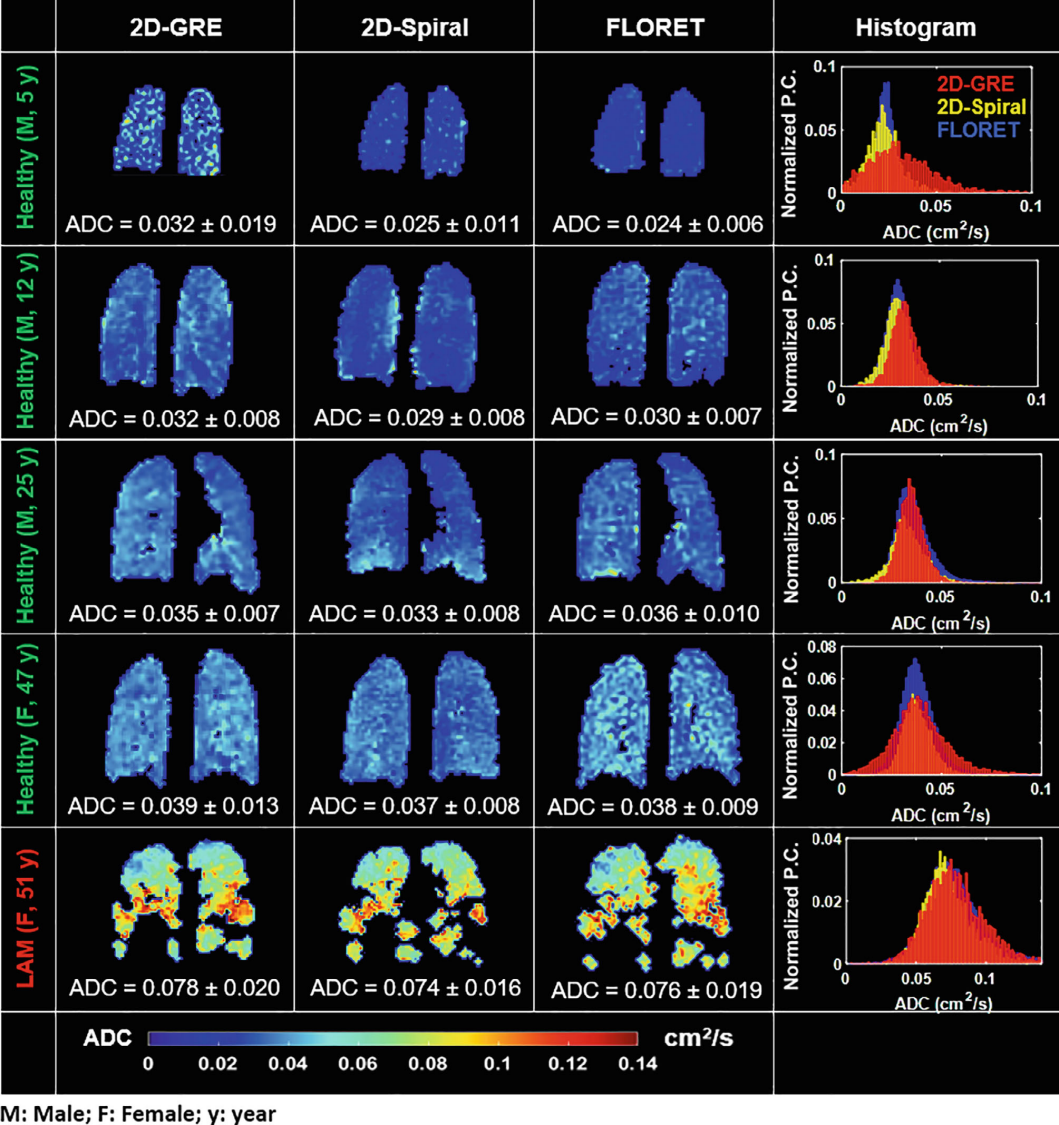
Representative slices from ADC maps of all subjects and sequences: First column, 2D‐GRE; second column, 2D‐Spiral; and third column, FLORET. The fourth column shows corresponding ADC histograms (Pixel count [P.C.] is normalized to account for the large number of voxels [˜10‐fold larger than 2D‐GRE or 2D‐Spiral] acquired in FLORET). No obvious difference in mean ADC or distribution was observed between the three sequences. Note that, the 5 y old subject could not hold his breath to the end of the 2D‐GRE scan. Exhalation resulted in lower SNR, 0.5‐fold higher mean ADC, and a one‐fold wider ADC distribution.

Representative ADC maps from all subjects and pulse sequences are shown in Figure 6. All three sequences show minimal differences in whole‐lung, mean ADC (difference < ±10%, *P* > 0.54) for individual subjects who fully complied with breath‐hold maneuver. For healthy controls, the ADC distributions were similarly homogeneous (CV < 25%) across sequences. Relative to age matched healthy subjects (Mean ADC = 0.023–0.04 cm^2^/s), mean ADC was elevated in the LAM patient (Mean ADC = 0.07 cm^2^/s), consistent with cystic alveolar destruction.^14^, ^20^

Negligible differences are seen in the shape or width of the ADC distributions obtained from healthy older subjects (<10% difference in means and SDs). However, the 5‐y‐old healthy subject was unable to hold his breath for the entire 16 s GRE scan duration. Lack of breath‐hold compliance resulted in GRE diffusion images with poor image SNR (<15) and more variable ADC values (SD‐ADC_2D‐GRE_ = 0.019 cm^2^/s). In contrast, the same subject was able to comply with the 4‐s breath‐hold for 2D‐Spiral, resulting in a narrower ADC distribution (SD‐ADC_2D‐Spiral_ = 0.011). Of note, the 5 y old subject was also unable to hold his breath until the end of the 15‐s FLORET scan, but the FLORET images displayed high $\mathrm{SNR}0_{\mathrm{EX}}$ ^18^ and yielded a narrower ADC distribution (SD‐ADC_FLORET_ = 0.006). This robustness to a non‐compliant breath‐holds likely results from FLORET sequence heavily oversampling k_0_, making it intrinsically robust to motion and similar forms of data degradation. Finally, the impact of non‐compliant breath‐holds (e.g., exhaling during scanning) is spread uniformly across the image in FLORET, whereas in 2D‐GRE is localized the effects to slices acquired during premature exhalation.

**FIGURE 7:**
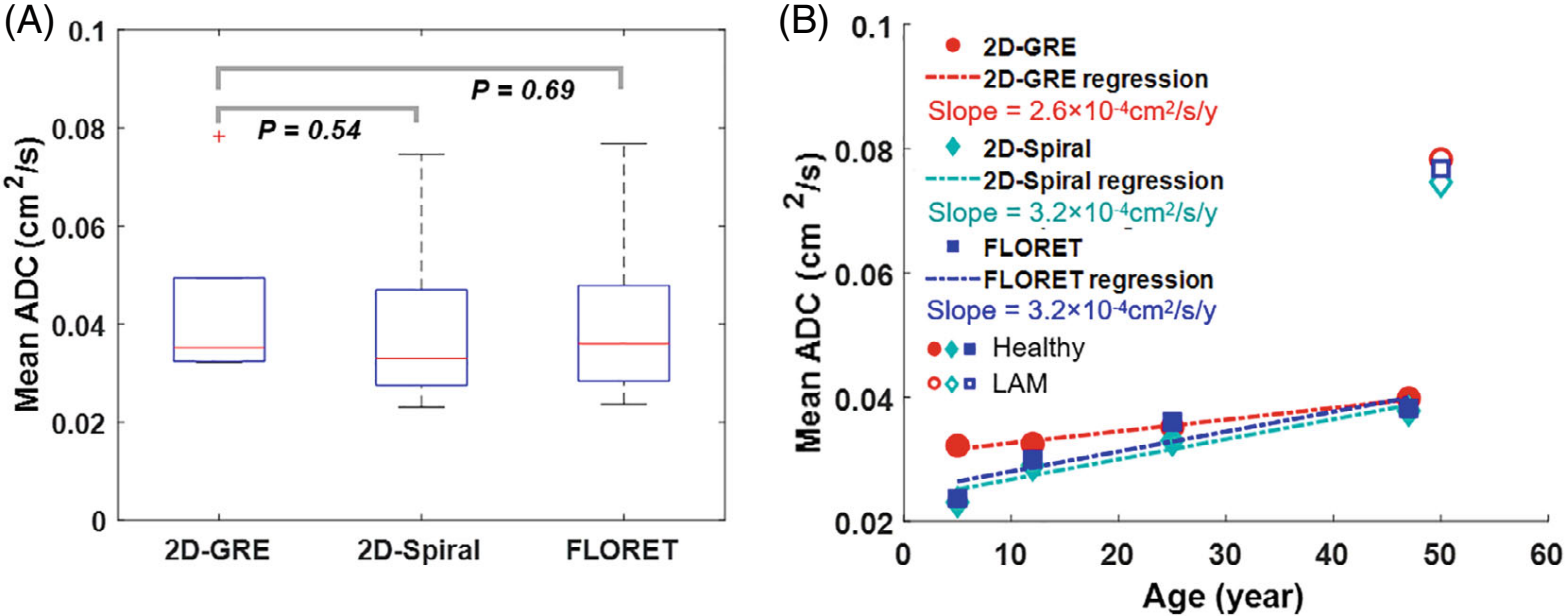
ADC comparison across sequences. A, Mean ADC across subjects and sequences. No significant difference in mean ADC across all subjects between the 2D‐GRE and 2D‐spiral (*P*_*mean*_ *= 0.54*) or FLORET (*P*_*mean*_ *= 0.69*). B, Mean ADC increased linearly with age (*R* ^*2*^ *> 0.82; P < 0.05*) for healthy subjects (i.e., excluding the LAM subject) as expected and showed no significant difference in best fit slopes (*P*_*rate*_ *>0.82*) between spiral and 2D‐GRE sequences. Additionally, the LAM subject displayed a nearly two‐fold higher mean ADC relative to a similarly aged healthy subject.

The similarity of ADC across sequences is further examined in Figure 7. Mean ADC across subjects (Figure 7A) was 0.043 ± 0.019 cm^2^/s, 0.039 ± 0.020 cm^2^/s and 0.040 ± 0.020 cm^2^/s when obtained with 2D‐GRE, 2D‐Spiral and FLORET, respectively, and there were no significant differences between sequences (*P = 0.54*). Additionally, mean ADC from healthy subjects varied significantly with age for all sequences (Figure 7B) (*R* ^*2*^ *> 0.82; P < 0.05*). However, there was no significant difference between best fit slopes (*P*_*rate*_ *>0.82*) across sequences. Consistent with cystic lung damage, the LAM subject produced higher whole‐lung mean ADC (0.075 cm^2^/s) relative to the similarly aged healthy control subjects.^11^, ^14^

### 4.5 Voxel‐level RF induced decay correction (2D‐spiral)

**FIGURE 8:**
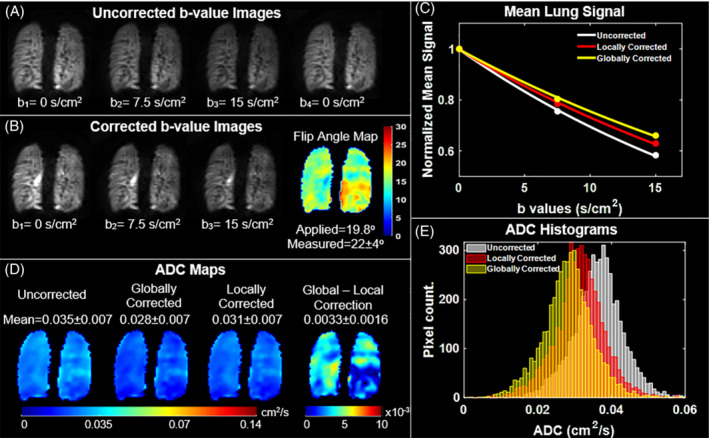
Correcting RF induced signal decay using 2D‐Spiral. A, Representative slice of the uncorrected *b*‐value images for a healthy subject (12 y old). B, Diffusion‐weighted images corrected using Eq. 9 and the flip angle map calculated from Eq. 8 (right side of panel) [The bright spot in the right lung is likely an airway]. C, Mean signal of the uncorrected, globally corrected and locally corrected images across the *b*‐values. D, ADC maps showing uncorrected, globally corrected, and locally corrected data. The corrections reduced the mean ADC by ˜22% and ˜12% with the global and local corrections, respectively. ADC difference map (global – local correction) is shown to the right and demonstrates overcorrection and an increase in mean ADC of 0.0033 ± 0.0016 cm^2^/s using global correction. E, Histograms of the uncorrected and globally and locally corrected ADC maps showing the ADC distribution is shifted to lower values and the mean is reduced by 0.007 and 0.004 cm^2^/s using the global and local corrections, respectively.

Figure 8 depicts the use of 2D‐Spiral diffusion mapping with a second b = 0 s/cm^2^ image to correct B_1_ inhomogeneity and RF‐induced signal decay via Eqs. 8 and 9. The mean of the measured flip angle maps was 22 ± 4°, which was 10% higher than the nominal flip angle of 19.8°, and similar to the difference observed for the second subject imaged using this approach (mean 24 ± 6° vs 20°, not shown). This led to the globally corrected mean ADC being reduced by 22% (0.029 ± 0.008 cm^2^/s), relat**i**ve to mean ADC of the uncorrected images (0.035 ± 0.008 staticm^2^/s), whereas the mean ADC yielded by correcting locally with the flip angle map was reduced by only 12% (0.031 ± 0.008 cm^2^/s). A similar pattern of over correction (0.023 ± 0.011 for global versus 0.025 ± 0.006 cm^2^/s for local correction) was observed in the second subject. Of note, these locally corrected means agree closely with the mean ADC obtained using GRE (0.032 ± 0.008 and 0.032 ± 0.019 cm^2^/s for the first and second subjects, respectively) and the FLORET (0.030 ± 0.007 and 0.023 ± 0.006 cm^2^/s), which used smaller flip angles (three‐ and five‐fold, respectively) and thus generated less RF‐induced decay across *b*‐values.

## 5 DISCUSSION

In this study, we applied 2D and 3D spiral (FLORET^29^, ^30^) for HP ^129^Xe diffusion imaging and compared the results to those obtained via conventional 2D‐GRE. Both 2D and 3D spiral acquisitions generated acceptable HP ^129^Xe image quality and ADC values that were comparable to those from conventional 2D‐GRE. Once corrected for RF‐induced signal decay (either globally or locally), the small differences (<10%) in mean ADC values across the three pulse sequences is similar to scan‐to‐scan variability using conventional GRE sequences.^47^, ^48^, ^49^

While 2D‐Spiral images displayed minor blurring artifacts, likely due to the longer readout (10 ms) used in these experiments, the sequence provided identical lung coverage to 2D‐GRE with three‐fold reduced acquisition times, thus enabling shorter breath‐holds. Furthermore, 2D‐Spiral generated higher image signal, because larger flip angles and fewer RF excitations were used. The theoretical SNR gain was not fully realized, due to noise‐like background artifacts. These artifacts were found primarily near high‐signal edges, and likely result from a combination of Gibbs ringing, long readout window, large flip angle, or off‐resonance effects. Despite these artifacts, the 2D‐Spiral pulse sequence still provided image $\mathrm{SNR}0_{\mathrm{EX}}$ >15, which is sufficient to provide accurate estimates of ADC.^14^, ^24^ Moreover, these artifacts could be further mitigated using variable‐density k‐space sampling trajectories^50^ or through improved image reconstruction.

The in vivo T_1_ of ˜30 s^31^, ^32^ is long relative to the acquisition time for a given slice (2–5 s, using 2D‐GRE). However, this acquisition time is comparable to the longest breath‐holds needed for ADC measurements (˜15 s). For example, the in vivo acquisition parameters for 2D‐GRE used in this work, longitudinal magnetization (and thus SNR) was reduced by ˜40% for the final image slice. As a result, SNR of the slices acquired later in in the breath‐hold suffered reduced signal and increased ADC uncertainty.^14^ However, with the faster 2D‐Spiral acquisition times,^28^ the SNR would be similar for all slices, improving the accuracy of ADC measurements in the final slices acquired.

The 10‐ms acquisition times used in this work are comparable to the ˜18 ms $T_{2}^{*}$ decay of ^129^Xe in the lungs at 3T,^33^ and this is expected to degrade high‐frequency k‐space data to some degree. However, the ratio of the readout duration, $T_{s}$, to apparent transverse relaxation time, ${T_{s}/T}_{2}^{*}$ should be <1 (assuming no system imperfections, e.g., off‐resonance, etc.),^29^, ^30^ which is satisfied in our work. This allows for rapid spiral acquisitions, while maintaining reasonable SNR and true image resolution. Using FLORET, it will be difficult to further improve true resolution with current gradient performance, because scan time is already at the maximum comfortable breath‐hold duration. That is, reducing the sampling time and increasing spiral number would reduce blurring, but the matrix size would have to be reduced (i.e., voxel size increased) to accommodate a ˜15 s breath‐hold. In contrast, blurring could be further reduced in 2D‐Spiral by using an increased number of shortened spiral views at the expense of increasing scan time by only a few seconds.

For 2D‐GRE images, signal magnitude is dominated by a single, central line of k‐space, so RF‐induced decay is negligible across diffusion‐weightings. Thus, even with our largest flip angle (8.6°), RF attenuation across *b*‐values at k‐zero was <5%. Similarly, RF‐induced decay was minimal for FLORET, because smaller flip angles of 3.5–5° were used. In contrast, 2D‐Spiral used flip angles of 15°–25° to increase SNR, but this generated RF‐induced signal decay comparable to diffusive signal attenuation, thus spuriously increasing the estimated ADC. While diffusion‐images can be corrected using the prescribed flip angle, global correction does not account for regional differences due to B_1_ inhomogeneity. To mitigate the impact of B_1_ variation by enabling regional corrections, a second b = 0 s/cm^2^ image at the end of the acquisition was acquired. This modification increased scan time by <2 s and allowed flip angle to be mapped and image signal intensity to be corrected voxel‐by‐voxel without sacrificing image resolution. Although not examined in this work, we expect similar improvements in accuracy of morphometric parameters to be obtained using this approach.

Finally, 2D‐Spiral allowed rapid slice acquisition, which will enable contiguous ADC mapping of the entire lung during a short breath‐hold (<6 s) with up to 4 *b*‐values. Contiguous ADC maps will be especially useful in early disease, in which alveolar damage begins as small, focal lesions disease^20^, ^23^ that could be missed using slice gaps. Moreover, complete lung coverage will be possible in subjects who cannot perform lengthy breath‐holds, including patients with severely compromised lung function and non‐compliant subjects (e.g., the 5‐y‐old imaged in this work). In contrast, breath‐hold times could be increased for compliant subjects to improve in‐plane resolution, reduce slice thickness, or enable shorter spiral readouts to reduce image blurring.

While FLORET requires a similar breath‐hold duration to 2D‐GRE (16 s), it is more robust to poor breath‐hold compliance. Furthermore, it yields complete lung coverage with isotropic (5 mm) resolution. Additionally, FLORET images seem to benefit from fewer artifacts and better noise background suppression. As such, this sequence is expected to be advantageous in applications where high resolution, high SNR, and/or full lung coverage is required.

### 5.1 Study limitations

Based on straightforward theoretical considerations, $\mathrm{SNR}0_{\mathrm{TH}}$ was expected to be higher for 2D‐Spiral and to be lower for FLORET sequences, relative to 2D‐GRE. However, this simple treatment (fixed BW, constant noise level, etc.) does not account for noise‐like contributions resulting from image acquisition and reconstruction imperfections (e.g., Gibbs ringing, artifacts from HP magnetization decay, etc.). Furthermore, sampling efficiency, which is 1 for GRE and <1 for all non‐Cartesian sequences, was not addressed.^30^ Therefore, a more rigorous model that accounts for these factors would be required for a complete, analytical evaluation of SNR across sequence types and experimental parameters.

2D‐Spiral enables shortened breath‐hold durations with the ability to obtain local flip‐angle and correct RF‐induced signal decay by acquiring a second *b*‐value = 0 image, thus increasing the accuracy of ADC. However, the paired *b*‐value image approach calculates each image from an interleaved set of spirals/*b*‐values (see Figure 1B). This could lead to higher uncertainty in the measured flip angle map which limits the use of this approach in low image SNRs. ^51^ However, future work could involve using Keyhole‐reconstruction techniques,^35^ which can be used to extract the flip angle maps with no additional data. This will result in less uncertainty and more accurate evaluation of flip angle variation over space, thus generating more accurate diffusion estimates without the need to acquire additional data.

## 6 CONCLUSIONS

Diffusion‐weighted hyperpolarized ^129^Xe MRI is a validated measure of lung microstructure and can noninvasively assess changes in alveolar dimensions. These images are commonly acquired via 2D‐GRE, and typically suffer from coarse in‐plane resolution, thick slices and incomplete lung coverage. Furthermore, the required long breath‐hold durations (≤16 s) may be difficult to perform for pediatric and/or severely ill subjects. To overcome these limitations, we implemented ^129^Xe diffusion imaging with efficient 2D and 3D (FLORET) spiral sequences. These sequences display image quality and ADC accuracy comparable to that of conventional 2D‐GRE, thus enabling either rapid acquisition or high‐resolution, isotropic lung coverage. Moreover, with relatively modest changes to the experimental protocol, it is possible to correct RF‐induced signal decay and generate more accurate diffusion estimates.
